# sgRNA-shRNA Structure Mediated SNP Site Editing on Porcine *IGF2* Gene by CRISPR/StCas9

**DOI:** 10.3389/fgene.2019.00347

**Published:** 2019-04-18

**Authors:** Yongsen Sun, Nana Yan, Lu Mu, Bing Sun, Jingrong Deng, Yuanyuan Fang, Simin Shao, Qiang Yan, Furong Han, Zhiying Zhang, Kun Xu

**Affiliations:** Key Laboratory of Animal Genetics, Breeding and Reproduction of Shaanxi Province, College of Animal Science and Technology, Northwest A&F University, Yangling, China

**Keywords:** *IGF2* gene, SNP, base editing, CRISPR, StCas9, sgRNA-shRNA

## Abstract

The SNP within intron 3 of the porcine *IGF2* gene (G3072A) plays an important role for muscle growth and fat deposition in pigs. In this study, the StCas9 derived from *Streptococcus thermophilus* together with the Drosha-mediated sgRNA-shRNA structure were combined to boost the G to A base editing on the *IGF2* SNP site, which we called “SNP editing.” The codon-humanized StCas9 as we previously reported was firstly compared with the prevalently used SpCas9 derived from *Streptococcus pyogenes* using our idiomatic surrogate report assay, and the StCas9 demonstrated a comparable targeting activity. On the other hand, by combining shRNA with sgRNA, simultaneous gene silencing and genome targeting can be achieved. Thus, the novel IGF2.sgRNA-LIG4.shRNA-IGF2.sgRNA structure was constructed to enhance the sgRNA/Cas9-mediated HDR-based *IGF2* SNP editing by silencing the *LIG4* gene, which is a key molecule of the HDR’s competitive NHEJ pathway. The sgRNA-shRNA/StCas9 all-in-one expression vector and the IGF2.sgRNA/StCas9 as control were separately used to transfect porcine PK15 cells together with an ssODNs donor for the *IGF2* SNP editing. The editing events were detected by the RFLP assay, Sanger sequencing as well as Deep-sequencing, and the Deep-sequencing results finally demonstrated a significant higher HDR-based editing efficiency (16.38%) for our sgRNA-shRNA/StCas9 strategy. In short, we achieved effective *IGF2* SNP editing by using the combined sgRNA-shRNA/StCas9 strategy, which will facilitate the further production of base-edited animals and perhaps extend for the gene therapy for the base correction of some genetic diseases.

## Introduction

The CRISPR/Cas9 technology (Jinek et al., 2012; Mali et al., 2013) has been widely used for genome editing in various cell types and organisms since its advent. So far, the *S. pyogenes*-derived SpCas9 is the most prevalently applied Cas9 enzyme (Cong et al., 2013). Nevertheless, Cas9 variants from different microbial species can also contribute efficient genome editing, such as *S. thermophilus* (StCas9) (Xu et al., 2015a) and *Staphylococcus aureus* (SaCas9) (Kleinstiver et al., 2015; Ran et al., 2015).

The Cas9 endonuclease is directed by an artificial single-guide RNA (sgRNA or gRNA) to recognize specifically the target DNA sequence with given protospacer adjacent motif (PAM) by base pairing (Jinek et al., 2012). In mammalian cells, the DNA double-strand breaks (DSBs) induced by the endonucleases can be repaired by two main mechanisms, the error-prone non-homologous end joining (NHEJ) and the donor-dependent homology-directed repair (HDR). The NHEJ pathway generates stochastic nucleotide insertions and deletions (Indels) at the target locus resulting in open reading frame (ORF) shift and loss-of-function of target genes. Alternatively, the HDR pathway can result in desired genome editing events by targeted recombination of designed homologous DNA template donors (Salsman and Dellaire, 2017).

In animal breeding researches, researchers usually knock-out/in genes of interest (GOI) to study their function and relationship in the network of signaling pathways (Komor et al., 2017). However, it’s always confronted with the concern of genetic safety, when creating gene knock-out/in animals. On the other hand, precise genome editing takes the advantage of the HDR pathway to make point mutations for gene function study, as well as gene therapy and animal breeding researches (Komor et al., 2017; Salsman and Dellaire, 2017). However, the HDR efficiency is extremely lower than NHEJ in mammalian cells. Hence, different approaches have been reported to enhance the efficiency of the HDR-based precise genome editing. Firstly, inhibiting the key molecules of the competitive NHEJ pathway, such as DNA ligase IV (LIG4) and KU70 (Maruyama et al., 2015; Hu et al., 2018); Secondly, optimizing the DNA template donors (ssDNA or dsDNA); Thirdly, several molecules or small compounds have been also reported to improve the HDR efficiency significantly, such as RAD51 and RAD52 (homologous recombination related proteins), Nocodazole and CCND1 (synchronizes cell cycle at specific phase) (Lin et al., 2014; Chu et al., 2015; Salsman and Dellaire, 2017; Shao et al., 2017).

We have previously developed the novel Drosha-mediated sgRNA-shRNA structure for transcribing multiple sgRNAs to promote the CRISPR/Cas9-based multiplex genome targeting (Yan et al., 2016). Interestingly, we noticed that the by-product shRNA could be used for silencing the *LIG4* gene to aid the HDR-based genome editing. Insulin like growth factor-2 (*IGF2*) is an important gene involved in pig muscle growth and fat deposition, and influencing the heart size. It has been reported that the NO.3072 nucleotide substitution from guanine (G) to adenine (A) within the intron 3 of the *IGF2* gene would obstruct the binding of the transcriptional inhibiting factor ZBED6, resulting in increased *IGF2* expression and muscle yield (Van Laere et al., 2003; Markljung et al., 2009). Our previous research has confirmed that it’s the wild-type G at the *IGF2* SNP site in the local pig species (Shao et al., 2017).

In this study, the codon-humanized StCas9 derived from *S. thermophilus* (Xu et al., 2015a) and the novel sgRNA-shRNA structure (Yan et al., 2016), both as we previously reported, were combined for enhancing the SNP editing of the porcine *IGF2* gene. Taking the advantage of the sgRNA-shRNA structure, simultaneous *IGF2* genome targeting by sgRNA/Cas9 and transient *LIG4* gene silencing by shRNA could be achieved. On the other hand, the CRISPR/StCas9 system with a stricter PAM requirement of NGGNG could reduce the off-target events compared with the CRISPR/SpCas9 system with the PAM pattern of NGG (Muller et al., 2016). Besides, the SNP editing also provided a good idea to avoid the genetic safety problem for the animal breeding study. Therefore, our novel sgRNA-shRNA/StCas9 strategy is of clear significance for gene editing or base correction, which will facilitate the further animal breeding research and the gene therapy study for the correction of genetic mutations.

## Materials and Methods

### Construction of sgRNA/Cas9 Expression Vectors and Surrogate Reporters

The IGF2.sgRNA/StCas9 expression vector (pll3.7-mU6-IGF2.sgRNA-CMV-hStCas9) was constructed with a further modified sgRNA scaffold as shown in Supplementary Figure S1, which was designed by referencing our (Xu et al., 2015a) and another (Chen et al., 2013) previous studies for optimizing the sgRNA structure. The IGF2.sgRNA/SpCas9 expression vector was subsequently constructed by replacing the CMV-StCas9 cassette of the IGF2.sgRNA/StCas9 vector with the CBh-SpCas9 cassette amplified from the plasmid pX330-U6-Chimeric_BB-CBh-hSpCas9 (Addgene, #42230).

A series of single strand annealing (SSA)-based surrogate reporters have been developed in our previous studies (Ren et al., 2019). The DsRed-eGFP (RG) and eGFP surrogate reporters (Xu et al., 2015a) were firstly designed and used for sgRNA/Cas9 activity verification in mammalian cells. The DsRed-Puro^R^-eGFP (RPG) surrogate reporter with dual-reporter genes was further constructed to assist the enrichment and screening of genetically modified cells by either puromycin selection or fluorescence-activated cell sorting (FACS) (Ren et al., 2015). The IGF2.RG and IGF2.eGFP surrogate reporters were constructed in this study as we previously did (Xu et al., 2015a), while the IGF2.RPG surrogate reporter was constructed in our previous study (Shao et al., 2017).

### Cell Culture and Transfection

The human embryonic kidney 293T (HEK293T) and porcine kidney epithelial (PK15) cells were cultured routinely in DMEM supplemented with FBS, penicillin and streptomycin as we previously did (Shao et al., 2017), with an additional 250 ng/ml antimycotic amphotericin B supplemented. The transfection assays were conducted within six-well plates using Lipofectamine^TM^ 2000 reagent (Invitrogen) following the manufacturer’s protocol, with a total of 3 μg plasmid DNA peer well. At least three independent wells were used for parallel transfections for each experiment group.

### Surrogate Report Assay for Comparing StCas9 and SpCas9

The human embryonic kidney 293T cells were co-transfected with the IGF2.sgRNA/StCas9 or IGF2.sgRNA/SpCas9 expression vector and the IGF2.RG surrogate reporter within six-well plates. At least three wells were used for parallel transfections for each group, and the molecular ratio for the sgRNA/Cas9 vector and the RG reporter was 1:1. After transfected for 2 days, the cells from each parallel well were harvested independently for flow cytometric analysis to count the DsRed^+^ single and DsRed^+^eGFP^+^ dual positive cells. The flow cytometric data was analyzed by the flowJo v10 software. The percentage of dual-fluorescence positive cells as DsRed^+^eGFP^+^/(DsRed^+^eGFP^+^+DsRed^+^) was calculated to evaluate the Cas9 activity indirectly.

### shRNA Design and Verification for Porcine LIG4 Gene Interference

Before designing the shRNAs against the porcine *LIG4* gene, three overlapping fragments (Supplementary Figure S2) from its complete CDS were amplified by RT-PCR with the PK15 cDNA as the template. The primers used are shown in Supplementary Table S1. The three PCR fragments were then sequenced and matched for verifying the sequence information of the *LIG4* gene. Afterward, three shRNA candidates were forecasted accordingly through the Invitrogen BLOCK-iT^TM^ RNAi Designer and the corresponding oligonucleotides as shown in Supplementary Table S2 were synthesized (Invitrogen). The three shRNA-1/2/3 cassettes were then generated by oligonucleotides-annealing (Xu et al., 2015b) and were cloned into the pLenti-H1 expression vector respectively. The non-specific shRNA vector pLenti-H1-SC (shRNA control) had been constructed previously in our lab. The H1-shRNA-CMV-eGFP expression cassettes from these four pLenti-H1 vectors were further amplified by PCR and cloned into the pB-CBh-puro vector. In addition to the fluorescent *eGFP* marker gene, the upgraded shRNA vectors (pB-CBh-Puro-H1-shRNA-CMV-eGFP) contained the puromycin resistant gene *(Puro^R^*) (Figure 2A), which was intent for the enrichment of the transfected cells by puromycin selection. The PK15 cells were transfected with these pB-based shRNA vectors. 24 h after the transfection, the cells were selected by puromycin (3 μg/ml) for about another 2 days, and then were harvested for the total RNA preparation and quantitative RT-PCR analysis.

### Quantitative RT-PCR Assay for LIG4 Gene Expression

Quantitative RT-PCR (qRT-PCR) assays were conducted as we previously performed (Yan et al., 2016) for detecting the relative transcript level of the *LIG4* gene. Generally, the parallel wells of transfected PK15 cells for each experiment or control group were collected independently for the total RNA isolation, the first-strand cDNA preparation and the further quantitative PCR analysis. The porcine β*-actin* gene was used as the internal control. The primers used for the qRT-PCR assays were listed in Supplementary Table S1.

### sgRNA-shRNA Structure Design and Activity Verification

We have reported the novel Drosha-mediated sgRNA-shRNA structure for multiplex genome targeting in previous study (Yan et al., 2016). Here, the LIG4.shRNA-1, which showed the highest activity for silencing the porcine *LIG4* gene in the preceding experiment, was used for the sgRNA-shRNA structure design. For further improvement, a pair of more efficient Drosha-processing sequences from miR-30 (Zeng and Cullen, 2005) were used to replace the former Drosha recognition sites. As designed in Figure 3A, the LIG4.shRNA sequence flanked by the Drosha-processing sequences (as shown in Supplementary Figure S3) was synthesized directly and inserted into the middle of two identical IGF2.sgRNA sequences. And then the IGF2.sgRNA-LIG4.shRNA-IGF2.sgRNA cassette was cloned into the pll3.7-mU6-CMV-hStCas9 vector (Xu et al., 2015a), generating the sgRNA-shRNA/StCas9 all-in-one expression vector, which was supposed capable for the simultaneous *IGF2* gene targeting and *LIG4* gene silencing.

Surrogate reporter assay was conducted to verify the sgRNA activity as described above. In brief, HEK293T cells were co-transfected with the IGF2.eGFP surrogate reporter and the sgRNA-shRNA/StCas9, the IGF2.sgRNA/StCas9, or the single StCas9 (pll3.7-mU6-CMV-hStCas9 with no sgRNA as the negative control) expression vector. The linearized IGF2.eGFP surrogate reporter, which was supposed to repair spontaneously in cells after the transfection, was used as the positive control to co-transfect the cells with the single StCas9 expression vector as we previously did (Xu et al., 2015a). After transfected for 2 days, the cells were photographed and cells from each parallel well were harvested independently for flow cytometric analysis. The percentage of eGFP positive cells was used as an indirect measurement for the IGF2.sgRNA activity.

In addition to the IGF2.sgRNA-LIG4.shRNA-IGF2.sgRNA (Sg-Sh) cassette, an IGF2.sgRNA-SC-IGF2.sgRNA (Sg-SC) cassette was generated by replacing the LIG4.shRNA-1 with the non-specific shRNA control. To verify the shRNA activity driven by the sgRNA-shRNA structure, the Sg-SC cassette, as well as Sg-Sh, was further cloned into the pLenti-H1 vector. Then, the pLenti-H1 based shRNA control (SC), LIG4.shRNA-1(Sh-1), Sg-SC and Sg-Sh expression vectors were used to transfect the PK15 cells, respectively, along with the pB-CBh-Puro vector. The transfected cells were enriched by puromycin selection for about 2 days as above, and then were harvested for detecting the relative expression of the *LIG4* gene by qRT-PCR analysis.

### Genome Editing of the Porcine IGF2 Gene

To conduct the HDR-based *IGF2* SNP editing, an 110 nt single-stranded oligodeoxynucleotides (ssODNs) with the desired G > A substitution (Supplementary Table S2) was synthesized by GenScript (Nanjing, China). The PAM motif of the IGF2.sgRNA target site within the ssODNs donor was mutated to the *Nhe*I restriction endonuclease (RE) site for the subsequent restrictive fragment length polymorphism (RFLP) assay.

Porcine kidney epithelial cells were co-transfected with the sgRNA-shRNA/StCas9 or IGF2.sgRNA/ StCas9 expression vector, the IGF2.RPG surrogate reporter and the ssODNs HDR donor. The transfections were conducted within six-well plates and the molecular ratio for RNA/Cas9:RPG reporter:ssODNs donor was 2:1:1. At least three wells were used for parallel transfections for each group. 2 days after the transfection, the cells were transferred into 60 mm dishes and were maintained continuously with puromycin treatment for another 5 days. After the puromycin selection, the resistant cell clones from the parallel dishes for each experiment group were collected as a pool as we previously did (Shao et al., 2017). The genomic DNAs for different pools were extracted, respectively, and the target locus was amplified by PCR for the subsequent RFLP and sequencing detections. The primers used were shown in Supplementary Table S1.

For the RFLP assay, the PCR products of the *IGF2* locus were 1319 bp in length. When the *IGF2* gene was edited successfully as designed, the PCR product would be cut into two fragments (1081 and 238 bp) by the *Nhe*I RE induced. On the other hand, the PCR products from the two experimental groups were further cloned into the pMD19-T “T-A” cloning vector, respectively, and a total of 40∼50 clones were picked for each group for Sanger sequencing. Simultaneously, the *IGF2* locus was also amplified for the Deep-sequencing analysis.

### Deep Sequencing Analysis

PCR amplicons of the two experiment groups were amplified, respectively, using different barcode-primer pairs (Supplementary Table S1), purified using a gel extraction kit (OMEGA Bio-Tek, China), and sequenced on an Illumina HiSeq (GENEWIZ, China). Among the files provided by GENEWIZ, reads with the sequence CTCaCAGCGCGctAGC (harboring both the desired G > A mutation and the PAM mutations) were considered as the HDR-based editing, and the percentage of HDR reads relative to all reads was calculated as the HDR efficiency for each group. The deep sequencing data are available under the BioProject ID: PRJNA526113.

### Statistical Analysis

For the histograms except Figure 4H, the data were collected from three independent experiments, and were analyzed by an unpaired and two-tailed *t*-test. Differences were considered statistically significant (^∗^) when *P* < 0.05. Error bars represented the standard error.

## Results

### StCas9 Showed Similar Activity With SpCas9

**FIGURE 1:**
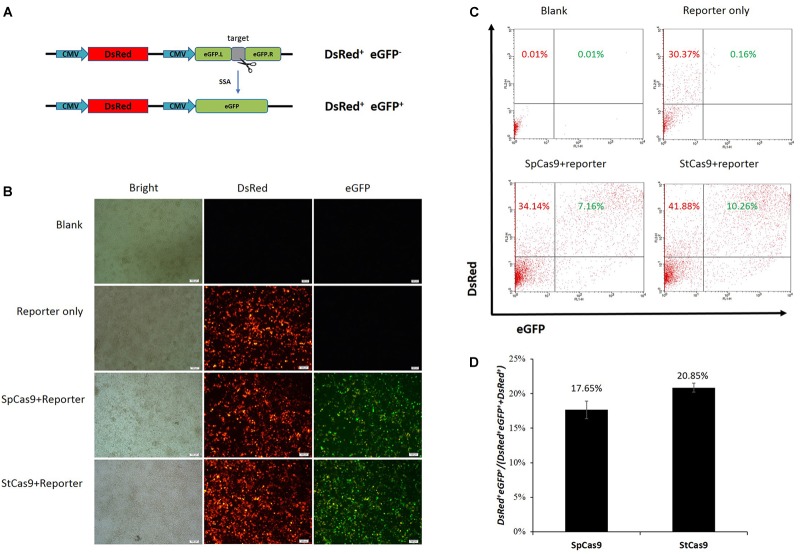
Surrogate report assay for comparing StCas9 and SpCas9. **(A)** The schematic of the SSA-based DsRed-eGFP (RG) surrogate reporter. The *DsRed* gene was used as the transfection marker, and the *eGFP* gene interrupted by the sgRNA target was used as the reporter, which was designed to be repaired accurately by SSA when targeted by the sgRNA/Cas9 complex (Xu et al., 2015a). **(B)** Representative visualization of the DsRed and eGFP fluorescence positive cells. The Cells were observed and photographed by fluorescence microscope 2 days after transfection. *Scale bar*, 100 μm. **(C)** Representative results of the flow cytometric counting analysis for the fluorescence positive cells. 20000 cells for each sample were counted to identify the DsRed^+^ and DsRed^+^ eGFP^+^ positive cells. **(D)** Comparison of the activities of SpCas9 and StCas9. The percentage DsRed^+^eGFP^+^/(DsRed^+^eGFP^+^+DsRed^+^ was calculated as an indirect measurement for the sgRNA/Cas9 activity. The data was analyzed by Student’s *t*-test (*n* = 3, *P* = 0.221).

We have developed the CRISPR/StCas9 system and the SSA-based DsRed-eGFP (RG) and eGFP surrogate reporters in our previous study (Xu et al., 2015a). Here, our codon-humanized StCas9 was firstly compared with SpCas9 using the dual-fluorescent RG surrogate report (Figure 1A). The *DsRed* gene was used as the transfection marker, and the interrupted *eGFP* gene was used as the reporter, which was designed to be repaired accurately by SSA when targeted by the sgRNA/Cas9 complex (Xu et al., 2015a). The HEK293T cells were observed under a fluorescent microscope 2 days after the transfection. Robust red fluorescence and obvious green fluorescence were evidenced within the cells from both SpCas9 and StCas9 experiment groups (Figure 1B). Then the cells were harvested and the DsRed^+^ single and DsRed^+^eGFP^+^ dual positive cells were counted by flow cytometric analysis (Figure 1C). The percentage of the DsRed^+^eGFP^+^ cells was calculated to evaluate the Cas9 activity, and our StCas9 demonstrated a comparable activity (20.85%) with the prevalently used SpCas9 (17.65%) (Figure 1D, *P* = 0.221).

### shRNA Verification for Porcine LIG4 Gene Interference

**FIGURE 2:**
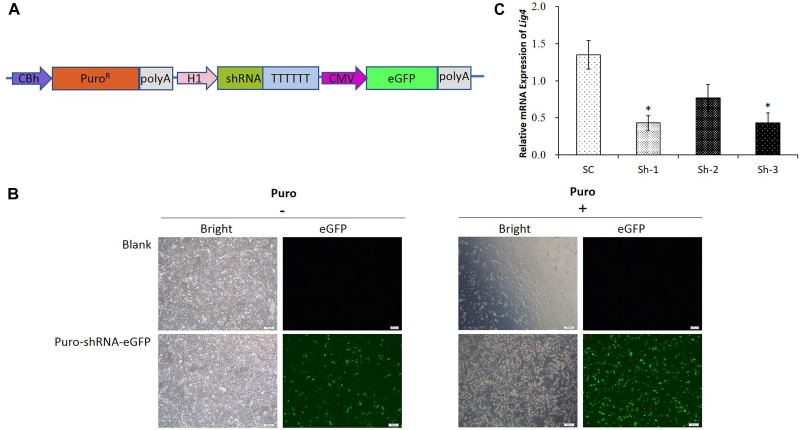
shRNA verification for porcine *LIG4* gene interference. **(A)** The diagrammatic drawing of the Puro^R^-shRNA-eGFP cassette, which contained the *Puro^R^* gene for the puromycin selection of transfected cells, as well as the fluorescent *eGFP* gene for the visualization. **(B)** Representative visualization of the un-transfected and transfected PK15 cells before or after the puromycin selection. The cells transfected with the shRNA vector were enriched significantly after the selection. **(C)** The relative expression of *LIG4* gene down-regulated by different shRNAs. The data was analyzed by Student’s *t*-test (*n* = 3, ^∗^*P* < 0.05 compared with SC). SC, the non-specific shRNA control.

Since the transfection efficiency for PK15 cells is limited, we constructed the upgraded shRNA vectors (Figure 2A), which contained the *Puro^R^* gene for the puromycin selection of the transfected cells, as well as the fluorescent *eGFP* gene for the visualization. The representative pictures for the un-transfected and transfected cells were shown in Figure 2B, which demonstrated that the PK15 cells transfected with the shRNA vector were enriched significantly after the puromycin selection. The results of the qRT-PCR analysis further confirmed that the relative expression of *LIG4* gene was declined about 60% by two of the shRNAs (Sh-1/3, Figure 2C, ^∗^*P* < 0.05 compared with SC).

### Functional Assay of the sgRNA-shRNA Structure

**FIGURE 3:**
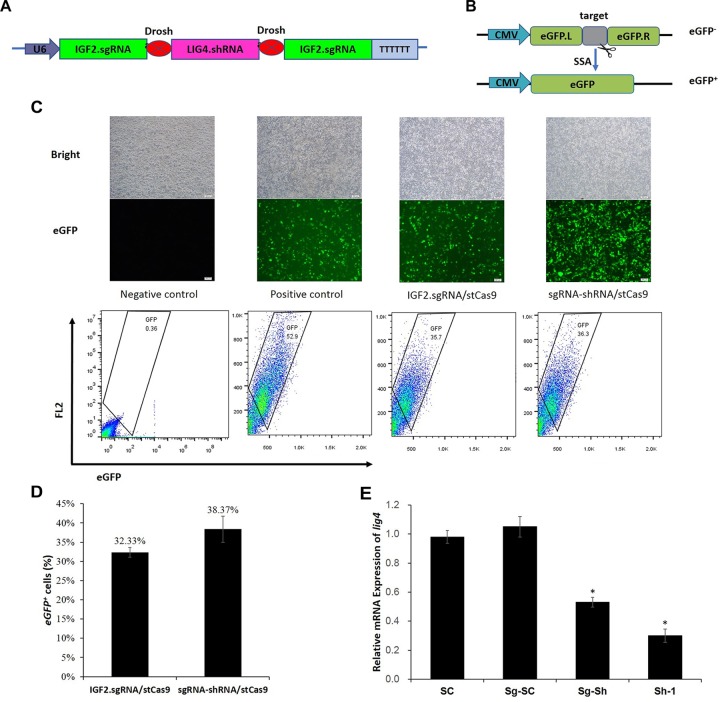
sgRNA-shRNA structure design and activity verification. **(A)** The sgRNA-shRNA structure was designed with two identical sgRNAs targeting the *IGF2* gene and one shRNA against the *LIG4* gene as we previously did (Yan et al., 2016). **(B)** The schematic of the SSA-based single-fluorescent eGFP surrogate reporter. The *DsRed* expression cassette was removed from the dual-fluorescent DsRed-eGFP (RG) surrogate reporter to avoid the interference of the robust red fluorescence on the green fluorescence reporter. **(C)** Representative pictures and flow cytometric counting results of eGFP^+^ cells. Negative control: cells transfected with IGF2.eGFP reporter and single StCas9 expression vector; Positive control: cells transfected with linearized IGF2.eGFP reporter and single StCas9 expression vector. **(D)** Comparison of the sgRNA activities driven by IGF2.sgRNA/StCas9 and sgRNA-shRNA/StCas9 (*n* = 3, *P* = 0.265). The percentage of eGFP^+^ cells was used as an indirect measurement for the IGF2.sgRNA activity. **(E)** The relative expression of *LIG4* gene down-regulated by different shRNAs or structures (*n* = 3, ^∗^*P* < 0.05 compared with SC). SC, the non-specific shRNA control; Sg-SC, sgRNA-shRNA structure with non-specific shRNA control; Sg-Sh, sgRNA-shRNA structure with LIG4.shRNA-1; Sh-1, LIG4.shRNA-1.

The sgRNA-shRNA structure was constructed with two identical sgRNAs targeting the *IGF2* gene and one shRNA against the *LIG4* gene (Figure 3A) as we previously did (Yan et al., 2016). The adjacent sgRNA and shRNA was linked by the optimized Drosha cutting sequences (Supplementary Figure S3).

Alternatively, the surrogate reporter assay was conducted using the single-fluorescent IGF2.eGFP reporter (Figure 3B) for verifying the sgRNA activity. The linearized IGF2.eGFP surrogate reporter, which was supposed to repair spontaneously in cells after the transfection, was used as the positive control to co-transfect the cells with the single StCas9 expression vector. The representative pictures of fluorescent cells and the flow cytometric counting results for different experiment groups were shown in Figure 3C. The percentage of eGFP^+^ cells was used as an indirect measurement for the IGF2.sgRNA activity. As shown in Figure 3D, the IGF2.sgRNA driven by the sgRNA-shRNA/StCas9 all-in-one expression vector (38.37%) demonstrated similar activity with that driven by the IGF2.sgRNA/StCas9 vector (32.33%, *P* = 0.265). To further verify the LIG4.shRNA activity driven by the sgRNA-shRNA structure, the qRT-PCR analysis was performed with both the SC (non-specific shRNA control) and Sg-SC (sgRNA-shRNA with non-specific shRNA) negative controls. The results suggested that the relative expression of *LIG4* gene was declined about 50% by the sgRNA-shRNA structure with LIG4.shRNA (Sg-Sh, Figure 3E, ^∗^*P* < 0.05 compared with SC).

### Efficient IGF2 Gene Editing by sgRNA-shRNA/StCas9

**FIGURE 4:**
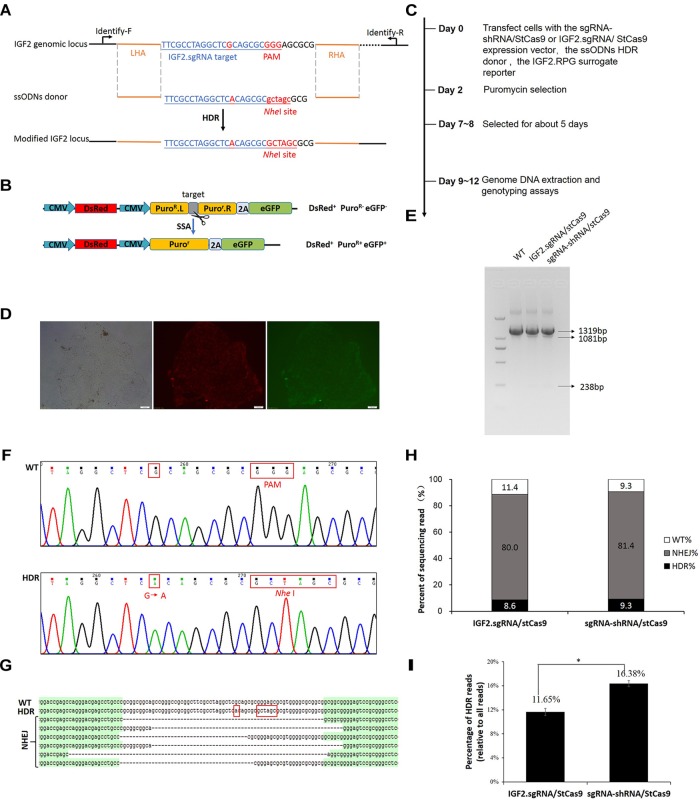
Efficient *IGF2* gene editing by sgRNA-shRNA/StCas9. **(A)** Schematic diagram for the HDR-based *IGF2* gene editing. The ssODNs donor was designed with the desired G > A substitution, as well as the mutations within the PAM motif. The *Nhe*I RE site was introduced for the subsequent RFLP assay. **(B)** The schematic of the SSA-based DsRed-Puro^R^-eGFP (RPG) surrogate reporter, which was used for the enrichment and screening of the targeted positive cells by puromycin selection (Ren et al., 2015). **(C)** Overview of the procedure for *IGF2* gene editing. **(D)** Representative porcine PK15 cell clone screened by puromycin selection. The green and red fluorescence were generated by the expression of the *DsRed* marker gene and the restored *eGFP* reporter gene within the RPG surrogate reporter. **(E)** RFLP assay for confirming the editing events at the *IGF2* locus. For the RFLP assay, the PCR products (1319 bp) of the *IGF2* locus would be cut into two fragments (1081 and 238 bp) by *Nhe*I when the *IGF2* gene was edited successfully as designed. **(F)** Representative chromatograms from the Sanger sequencing analysis for confirming the HDR-based editing events. The designed G > A substitution and PAM mutation (Red border) were introduced successfully. **(G)** Representative sequences of the *IGF2* locus with the HDR-based or NHEJ-based edits from the Sanger sequencing analysis. **(H)** Statistical analysis of the HDR-based and NHEJ-based edits from the Sanger sequencing analysis. **(I)** Statistical analysis of the reads with the HDR-based edits from the Deep-sequencing results. The data was analyzed by Student’s *t*-test (*n* = 3, ^∗^*P* < 0.05).

The IGF2.RPG surrogate reporter was constructed and used for the selection of the genetically modified positive cells as we previously reported (Ren et al., 2015; Shao et al., 2017). PK15 cells were co-transfected with the sgRNA-shRNA/StCas9 or the IGF2.sgRNA/StCas9 expression vector and the ssODNs HDR donor (Figure 4A), along with the IGF2.RPG surrogate reporter (Figure 4B). After the puromycin selection (Figure 4C), the resistant positive cell clones (Figure 4D) were pooled and the genomic DNA was extracted for the subsequent RFLP and Sanger sequencing analyses. For the RFLP assay, the PCR products (1319 bp) of the *IGF2* locus would be cut into two fragments (1081 and 238 bp) by *Nhe*I when the *IGF2* gene was edited successfully as designed (Figure 4A,E). The editing events were firstly confirmed by the results of both the RFLP assay (Figure 4E) and Sanger sequencing (Figure 4F). The Sanger sequencing results of the “T-A clones” for the *IGF2* target locus (Figure 4G) further demonstrated 9.3% (4/43) HDR-based and 81.4% (35/43) NHEJ-based repair efficiencies driven by sgRNA-shRNA/StCas9, while the HDR-based and NHEJ-based repair efficiencies for the IGF2.sgRNA/StCas9 group were 8.6% (3/35) and 80.0% (28/35), respectively (Figure 4H). As limited clones were sequenced by Sanger sequencing, which may not reveal the real difference between the two experiment groups, we further conducted the Deep-sequencing analysis and the results finally demonstrated a significantly higher HDR-based editing efficiency (16.38%) for the sgRNA-shRNA/StCas9 group (Figure 4I, ^∗^*P* < 0.05).

## Discussion

The HDR-based genome editing holds great promise to the development of safe and highly precise approaches for gene therapy and animal breeding researches (Steyer et al., 2018). Recent years, massive efforts have been made to enhance the CRISPR/Cas9-mediated HDR efficiency. Here, we combined the StCas9 and the novel sgRNA-shRNA structure for boosting the HDR-based “SNP editing” of the porcine *IGF2* gene.

We have developed the *S. thermophilus*-derived CRISPR/StCas9 system for eukaryotic genome editing in our previous study (Xu et al., 2015a). Although the CRISPR/StCas9 system could share the same NGG PAM with the *S. pyogenes*-derived CRISPR/SpCas9 system, it required a stricter NGGNG PAM pattern for full activity (Xu et al., 2015a), which may contribute to reduce the off-target effect compared with the CRISPR/SpCas9 system (Muller et al., 2016). We found in our study that the key elements of our optimized sgRNA scaffold (Xu et al., 2015a) were almost the same with that of the CRISPR/SpCas9 system (Mali et al., 2013). A series of our subsequent applications also suggested that the two systems may share the same sgRNA structure. However, our StCas9 remained to be compared with the prevalently used SpCas9. To compare the targeting activities of the two Cas9 variants, we used the uniform and further modified sgRNA scaffold (Supplementary Figure S1), and the surrogate report assay was conducted using our idiomatic SSA-based RG surrogate reporter. We are glad to see that our StCas9 demonstrated comparable activity with the SpCas9 (Figure 1D).

We developed the novel Drosha-mediated sgRNA-shRNA structure mainly for the multiplex genome targeting at the beginning (Yan et al., 2016). However, taking the advantage of the combined sgRNA-shRNA structure, simultaneous genome targeting by sgRNA/Cas9 and transient gene silencing by shRNA could be achieved. Interestingly, we noticed that the by-product shRNA could be used for interfering *LIG4* gene to enhance the HDR-based genome editing. During the multiplex genome targeting assays, we found that the sgRNAs driven by the sgRNA1-shRNA-sgRNA2 structure showed lower activity than the independent sgRNA controls driven by routine sgRNA/Cas9 expression vectors. Hence, we used the sgRNA-LIG4.shRNA-sgRNA structure with identical sgRNAs flanking the LIG4.shRNA to guarantee the sgRNA activity for the enhanced HDR-based genome editing (Yan et al., 2016). In this study, we used the same strategy and further applied a pair of more efficient Drosha-processing sequences (Zeng and Cullen, 2005) for the processing of the short RNAs. The surrogate reporter and qRT-PCR assays demonstrated effective sgRNA and shRNA activities driven by sgRNA-shRNA/StCas9 (Figure 3D,E). Another point to explain, since we have compared the sgRNA-shRNA structures with LIG4.shRNA and non-specific shRNA for enhancing the HDR-based genome editing, this study was designed mainly focusing on the practical question whether our sgRNA-shRNA strategy (with LIG4.shRNA) is better than the routine single sgRNA strategy.

It is reported that numerous human genetic diseases were caused by single nucleotide mutation, such as the 878 G > A (*AVPR2* W293X) in X-linked Nephrogenic diabetes insipidus and the 1517 G > A (*FANCC* W506X) in Fanconi anemia (Cox et al., 2017). Moreover, there have been so many SNP sites found in livestock, and most of them are related to animal diseases or the production traits, such as the 3072 G > A in the porcine *IGF2* gene in this study. Thus, the SNP editing provides a good idea for the production of base-edited animals, as well as the gene therapy for the base correction of some genetic diseases.

In view of the unavoidable concern of off-target effect during the genome editing manipulation, our sgRNA-shRNA/StCas9 system still has room for improvement. It has been reported that paired Cas9-nickase (Cas9n) can be used for efficient genome editing with significant decreased off-target events (Ran et al., 2013). Hence, an improved sgRNA-shRNA/StCas9n system may be a good idea in the further study.

## Author Contributions

KX and ZZ conceived the research plans. YS, NY, LM, BS, JD, and YF performed the experiments. SS, QY, and FH provided related plasmids. YS, NY, and KX wrote the article. KX provided financial support.

## Conflict of Interest Statement

The authors declare that the research was conducted in the absence of any commercial or financial relationships that could be construed as a potential conflict of interest.
