# Supplementary material for: sgRNA-shRNA Structure Mediated SNP Site Editing on Porcine IGF2 Gene by CRISPR/StCas9
Source: Front Genet. 2019 Apr 18;10:347. doi: 10.3389/fgene.2019.00347 (PMC6482158; doi:10.3389/fgene.2019.00347)
Supplement: Supplementary file 1 [file Data_Sheet_1.pdf]

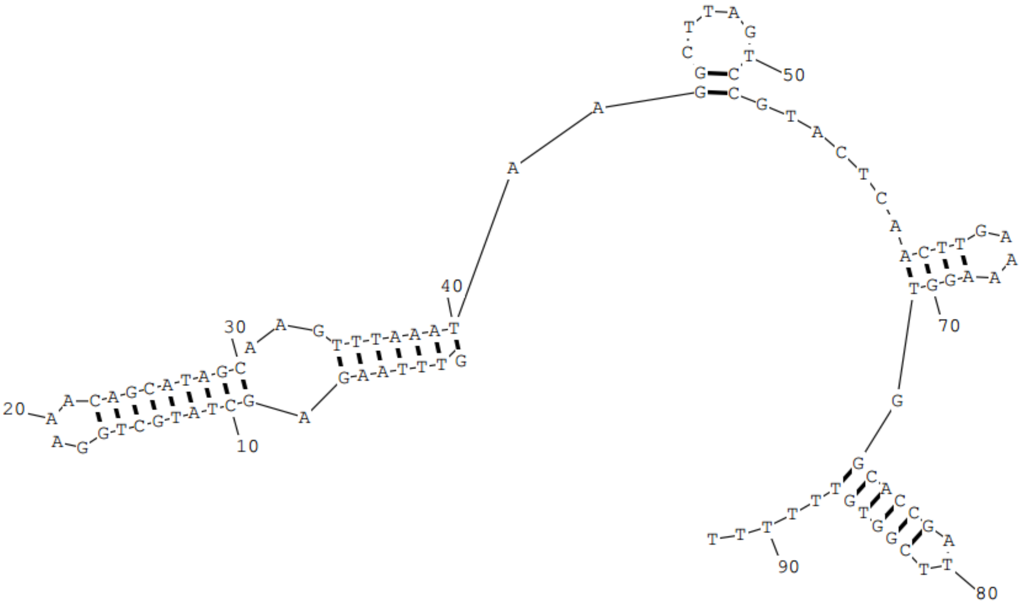

Fig.S1 Modified sgRNA scaffold for the construction of sgRNA/Cas9 vectors

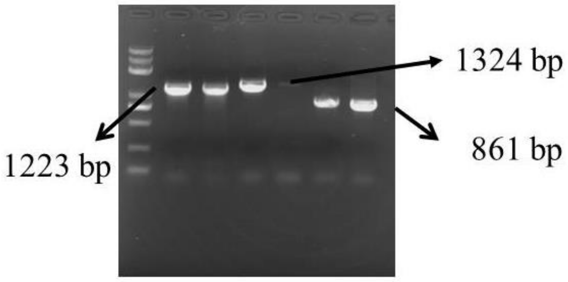

Fig.S2 Three overlapping *IGF2* CDS fragments amplified from PK15 cDNA

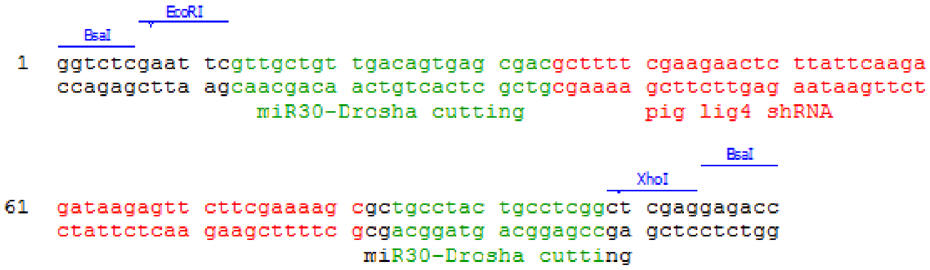

Fig.S3 Sequence map for the LIG4.shRNA flanked by the Drosha-processing sequences

Table S1 Primers used in this study

Name	Sequence (5′→3′)	Usage
LIG4.F1	AAGTCAGAAGATTCACTGTTGC	*LIG4* CDS cloning
LIG4.F2	TCCCGAAACGGATACAACT	
LIG4.F3	ACCTCAAAGCGCCTAACC	
LIG4.R1	TGTTGTAGTGAGTCAGCCAGAT	
LIG4.R2	TCTACCTGGTATGGGCGTA	
LIG4.R3	GCTGGGTCCTGAAACACTC	
H1-GFP.F	AActgcagATTTTCGGGTTTATTACAGGG	H1-shRNA-CMV-eGFP cloning
H1-GFP.R	CCttaattaaTTAGGTCCCTCGACGAAT	
IGF2.sgRNA.F	TTCGCCTAGGCTCGCAGCGCGGG	IGF2.sgRNA target annealing
IGF2.sgRNA.R	CCCGCGCTGCGAGCCTAGGCGAA	
LIG4.F	AAACCAAGCTAGACGGCGAAC	qRT-PCR analysis
LIG4.R	CCATGAATGAACGGGGTAAGG	
Pig.β-actin.F	ACTGGGACGACATGGAGAAGA	
Pig.β-actin.R	TTGGCTTTGGGGTTCAGG	
IGF2.Identify.F	CCCATCTCCCCCCCACCCCAT	Genotyping assays
IGF2.Identify.R	CACTCTGCCCTCTGATTCCTG	
Barcode24.F1	ggtagcAAAACTGGTTTCGCCCTCCTCCG	Deep sequencing
Barcode26.F2	atgagcTGTTGAAGTCCCCGAGAGCGCC	
Barcode27.F2	attcctTGTTGAAGTCCCCGAGAGCGCC	
Barcode25.R1	actgatGCCGACCGGGGAGCCTGG	
Barcode32.R1	cactcaGCCGACCGGGGAGCCTGG	

Note: The nucleotides in lowercase indicate restriction enzyme sites or the barcodes.

Table S2 Long oligonucleotides used in this study

Name	Sequence (5′→3′)
LIG4.sh1.F	gatccGCTTTTCGAAGAACTCTTATTCAAGAGATAAGAGTTCTTCGAAAAGCTTTTTc
LIG4.sh1.R	tcgagAAAAGCTTTTCGAAGAACTCTTATCTCTTGAATAAGAGTTCTTCGAAAAGCg
LIG4.sh2.F	gatccGGCTAAGAAGGTCATTGGATTCAAGAGATCCAATGACCTTCTTAGCCTTTTTc
LIG4.sh2.R	tcgagAAAAAGGCTAAGAAGGTCATTGGATCTCTTGAATCCAATGACCTTCTTAGCCg
LIG4.sh3.F	gatccGGCAGAAGTAGTGTCTCATTTCAAGAGAATGAGACACTACTTCTGCCTTTTTc
LIG4.sh3.R	tcgagAAAAAGGCAGAAGTAGTGTCTCATTCTCTTGAAATGAGACACTACTTCTGCCg
IGF2.ssODNs	AGCCAGGGACGAGCCTGCCCGCGGCGGCAGCCGGGCCGCGGCTTCGCCTAGGCTCaCAGCGCgctagcGCGTGGGGCGCGGCGGCGGCGGGGAGTCCGCGGGCCTCCTCG

Note: Three pairs of complementary oligonucleotides with overhangs “gatcc” (*BamHI*) and “tcgag” (*XhoI*) were used for constructing the pLenti-H1-LIG4.shRNA vectors. The loop sequences (TTCAAGAGA) are underlined. For IGF2 ssODNs donor, the G > A substitution and PAM mutation was surrounded by a border.
